# The Effectiveness of Pfizer-BioNTech and Oxford-AstraZeneca Vaccines to Prevent Severe COVID-19 in Costa Rica: Nationwide, Ecological Study of Hospitalization Prevalence

**DOI:** 10.2196/35054

**Published:** 2022-05-20

**Authors:** Luis Rosero-Bixby

**Affiliations:** 1 Centro Centroamericano de Población Universidad de Costa Rica San Pedro Costa Rica

**Keywords:** COVID-19, vaccine, effectiveness, hospitalization, epidemiology, prevention, severity, Costa Rica, observational, prevalence, real-world, virus, variant, policy, monitoring, surveillance

## Abstract

**Background:**

The Costa Rican COVID-19 vaccination program has used Pfizer-BioNTech and Oxford-AstraZeneca vaccines. Real-world estimates of the effectiveness of these vaccines to prevent hospitalizations range from 90%-98% for two doses and from 70%-91% for a single dose. Almost all of these estimates predate the Delta variant.

**Objective:**

The aim of this study is to estimate the dose-dependent effectiveness of COVID-19 vaccines to prevent severe illness in real-world conditions in Costa Rica, after the Delta variant became dominant.

**Methods:**

This observational study is a secondary analysis of hospitalization prevalence. The sample is all 3.67 million adult residents living in Costa Rica by mid-2021. The study is based on public aggregated data of 5978 COVID-19–related hospital records from September 14, 2021, to October 20, 2021, and 6.1 million vaccination doses administered to determine hospitalization prevalence by dose-specific vaccination status. The intervention retrospectively evaluated is vaccination with Pfizer-BioNTech (78%) and Oxford-AstraZeneca (22%). The main outcome studied is being hospitalized.

**Results:**

Vaccine effectiveness against hospitalization (VEH) was estimated as 93.4% (95% CI 93.0-93.9) for complete vaccination and 76.7% (95% CI 75.0-78.3) for single-dose vaccination among adults of all ages. VEH was lower and more uncertain among older adults aged ≥58 years: 92% (95% CI 91%-93%) for those who had received full vaccination and 64% (95% CI 58%-69%) for those who had received partial vaccination. Single-dose VEH declined over time during the study period, especially in the older age group. Estimates were sensitive to possible errors in the population count used to determine the residual number of unvaccinated people when vaccine coverage is high.

**Conclusions:**

The Costa Rican COVID-19 vaccination program that administered Pfizer-BioNTech and Oxford-AstraZeneca vaccines seems to be highly effective at preventing COVID-19–related hospitalization after the Delta variant became dominant. Even a single dose seems to provide some degree of protection, which is good news for people whose second dose of the Pfizer-BioNTech vaccine was postponed several weeks to more rapidly increase the number of people vaccinated with a first dose. Timely monitoring of vaccine effectiveness is important to detect eventual failures and motivate the public to get vaccinated by providing information regarding the effectiveness of the vaccines.

## Introduction

Concerns regarding the possible lack of effectiveness of a single dose of COVID-19 vaccine arose with the emergence of the Delta variant, a more contagious variant of COVID-19. In Costa Rica, COVID-19 cases caused by the Delta variant increased from 11% of new infections in the last week of June 2021 to 55% of new infections in the first week of August and to 100% in the last week of September 2021 (Instituto Costarricense de Investigación y Enseñanza en Nutrición y Salud [INCIENSA], unpublished data). In the same period, the incidence of COVID-19 increased from 288 to 445 daily cases per million population, despite the rapid increase in the proportion of vaccinated people from 32% on June 28, 2021, to 66% on September 27, 2021 [1].

The universal public health care system of Costa Rica, which is provided by the Costa Rican Social Security Fund (CCSS, acronym in the Spanish language), has been the single source of COVID-19 vaccination in the country. The CCSS uses two vaccines, the messenger RNA vaccine from Pfizer-BioNTech and the adenovirus vector vaccine from Oxford-AstraZeneca. By October 20, 2021, approximately 85% of the 3.7 million adult residents had been vaccinated with at least one dose and 59% with two doses [2]. Older adults aged ≥58 years received only the Pfizer-BioNTech vaccine; among older adults, 87% received the two doses with a 3-week interval and 13% with an 8- to 12-week interval. Among adults <58 years of age, 77% received the Pfizer-BioNTech vaccine and 23% received the Oxford-AstraZeneca vaccine, mostly with an 8- to 12-week interval between doses. The Costa Rican vaccination program had a strong initial focus on older adults (aged ≥58 years, as defined by the government). By June 1, 2021, more than 80% of the population in this age group had been vaccinated compared to 17% in the 40-57 years age group and 7% in the 20-39 years age group. Due to this initial focus on older adults, the proportion of older adults with more than a 6-month period after their second dose was growing rapidly during the study period, from 2% on September 14, 2021, to 24% on October 20, 2021.

At the time of this study, the literature reported the following real-world estimates (based on observational studies rather than randomized trials) of COVID-19 vaccine effectiveness against hospitalization (VEH). The 2-dose Pfizer-BioNTech VEH was 97% in Israel [3], 98% in Ontario, Canada, in a vaccination program that had primarily allocated Pfizer-BioNTech vaccine (77%) [4], 91% in the United States in the first 4 months after full vaccination [5], 90% in California when 93% of COVID-19 cases were caused by the Delta variant [6], and 90% in Qatar according to a preprint study on only Delta variant cases [7]. The highest VEH estimates (in Israel and Ontario) were obtained before the emergence of the Delta variant.

Single-dose VEH estimates were 70% in the Ontario vaccination program, which mostly used the Pfizer-BioNTech vaccine [4], 80% in England for both Pfizer-BioNTech and Oxford-AstraZeneca vaccines [8], and 91% and 88% in Scotland for Pfizer-BioNTech and Oxford-AstraZeneca vaccines, respectively [9]. All these estimates predated the Delta variant.

The objective of this study was to estimate the dose-dependent effectiveness of COVID-19 vaccines to prevent severe cases of COVID-19, as measured by the prevalence of hospitalizations, in a middle-income country (Costa Rica). These estimates were based on secondary analysis of COVID-19–related hospitalized individuals from September 14 to October 20, 2021.

## Methods

### Study Design

This observational, nationwide study used a cross-sectional prevalence design. The study performed secondary analysis of official statistics and reports. It compared the COVID-19–related hospitalization prevalence among the unvaccinated population with the prevalence among the semivaccinated and fully vaccinated populations at 6 time points, each 1 week apart, from September 14 to October 20, 2021.

### Data

The VEH estimates used three sources of data:

1. A series of weekly reports presented by the Department of Health Statistics to the Board of Directors of the CCSS (unpublished data), which is the most important information. These reports show the distribution by vaccination status of COVID-19–related hospitalizations by the ages of patients. CCSS officers linked the databases of hospitalizations and vaccinations to determine the vaccination status of hospitalized individuals and some demographic characteristics such as age and sex. For 2% of the hospital records, the vaccination status was not established.
2. The time series of the number of first and second doses of the COVID-19 vaccines administered (6.1 million by October 20, 2021) according to population age groups as reported weekly by the CCSS [2]. These data were used to estimate the nationwide populations of semivaccinated and fully vaccinated individuals by age group at the 6 time points of the study. No adjustments were made for changes in demographics (no vaccinated individual died, out-migrated, or changed age bracket) in these populations since the impact of these changes is small considering the short study period.
3. The mid-2021 nationwide population estimate by the National Institute of Statistics and Censuses (INEC) [10]. This estimate was used to determine the residual group of unvaccinated individuals, who represented the control group in the analysis. It was assumed that there were no changes in the population from the date of the estimate to the study dates.

### Variables

The outcome variable was “being hospitalized due to COVID-19.”

The intervention variables were the two vaccination statuses as defined by the CCSS:

1. Partially or semivaccinated individuals: 15 or more days after the first dose and either less than 15 days after the second dose or no second dose.
2. Fully vaccinated individuals: 15 or more days since the second dose.

All analyses were stratified according to three age groups: 20-39, 40-57, and ≥58 years. These age brackets were defined in the priority calendar of the national vaccination program.

### Statistical Methods

VEH is an epidemiological measure of relative risk reduction. Therefore, it was estimated as one minus the hospitalization prevalence ratio of vaccinated to unvaccinated populations.

Given the strong confounding effects of age, the Mantel-Haenzel technique was used to aggregate the age-specific estimates into a summary indicator for the entire adult population [11]. No imputations were made for the 2% of hospitalizations that had missing data, which were assumed to be randomly distributed. Estimates were obtained using Stata 17 statistical software (version 17; StataCorp LLC) and its “epitab” commands [12].

Although the number of vaccinated persons is a direct count of administered vaccines, the number of unvaccinated persons was an indirect estimate of the residual: population minus the number of vaccinated people. Errors in the population estimate would therefore overestimate or underestimate the number of unvaccinated individuals. A sensitivity analysis was performed to assess the impact of this potential error on the VEH.

### Ethics Statement

This study is a secondary analysis of aggregated public data and as such does not need clearance or permissions from an ethics committee.

## Results

### Participants

Overall, the study included data of 3.67 million individuals, the entire adult population of Costa Rica. Of this population, 47% were in the younger group, 31% in the intermediate group, and 22% in the older group. The number of hospital records assessed in the 6 time periods was 5978, excluding 138 records with missing information. Table 1 shows the data used in the study, namely the number of participants (the population) and COVID-19–related hospitalizations. The table also shows the resulting prevalence proportions.

The highest rates of hospitalization occurred among older unvaccinated individuals, with a prevalence of 3537-4765 per million people. The lowest rates of hospitalization occurred among fully vaccinated younger adults, with prevalence ranging from 7-32 per million people, approximately 400 times lower than unvaccinated older adults. Hospitalization prevalence increased with age and was substantially higher among unvaccinated individuals. Over time, the prevalence proportions reflected the fact that COVID-19 cases in Costa Rica had reached their peak at the beginning of September, followed by a peak in hospitalizations 2 weeks later [13].

**Table 1:** Population, number of COVID-19–related hospitalized people, and hospitalization prevalence by vaccination status and age at 6 points in time, using data from Costa Rica.

Date and vaccination status			Age 20-39 years							Age 40-57 years							Age ≥58 years				
			N-pop^a^		N-hosp^b^		Prev^c^		N-pop			N-hosp		Prev		N-pop			N-hosp		Prev
**September 14, 2021**																					
	Total	1,732,200		249		144		1,124,100			476		423		817,400			476		582	
	No	602,453		189		314		243,809			302		1239		63,883			302		4727	
	Semi	944,645		54		57		593,039			147		248		20,660			147		7115	
	Fully	185,102		6		32		287,252			27		94		732,857			27		37	
**September 22, 2021**																					
	Total	1,732,200		255		147		1,124,100			428		381		817,400			428		524	
	No	596,169		204		342		240,656			302		1255		63,374			302		4765	
	Semi	824,275		42		51		458,855			91		198		16,322			91		5575	
	Fully	311,756		9		29		424,589			35		82		737,704			35		47	
**September 29, 2021**																					
	Total	1,732,200		239		138		1,124,100			409		364		817,400			409		500	
	No	551,141		180		327		224,643			287		1278		62,052			287		4625	
	Semi	790,324		54		68		355,344			84		236		14,192			84		5919	
	Fully	390,735		5		13		544,113			38		70		741,156			38		51	
**October 6, 2021**																					
	Total	1,732,200		233		135		1,124,100			377		335		817,400			377		461	
	No	469,731		165		351		195,425			250		1279		58,864			250		4247	
	Semi	740,414		57		77		263,446			80		304		14,829			80		5395	
	Fully	522,055		11		21		665,229			47		71		743,707			47		63	
**October 13, 2021**																					
	Total	1,732,200		196		113		1,124,100			312		278		817,400			312		382	
	No	387,924		130		335		164,113			194		1182		53,971			194		3595	
	Semi	724,720		57		79		227,889			79		347		17,645			79		4477	
	Fully	619,556		9		15		732,098			39		53		745,784			39		52	
**October 20, 2021**																					
	Total	1,732,200		159		92		1,124,100			232		206		817,400			416		509	
	No	354,165		108		305		150,857			146		968		51,176			181		3537	
	Semi	704,530		46		65		208,980			52		249		18,588			31		1668	
	Fully	673,505		5		7		764,263			34		44		747,636			204		273	
**Pooled (averages)**																					
	Total	1,732,200		222		128		1,124,100			372		331		817,400			403		493	
	No	493,597		163		330		203,251			247		1214		58,887			253		4291	
	Semi	788,151		52		66		351,259			89		253		17,039			85		5008	
	Fully	450,452		8		17		569,591			37		64		741,474			65		88	

^a^N-pop: population.

^b^N-hosp: number of COVID-19–related hospitalized people.

^c^Prev: hospitalization prevalence per 1 million population.

### Vaccine Effectiveness

**Figure 1:**
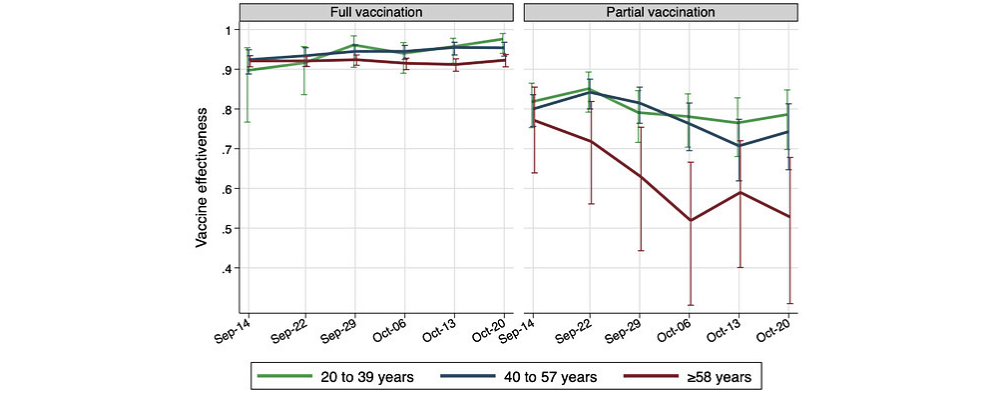
COVID-19 VEH estimates and their 95% CIs by age group and cross-section date, in Costa Rica, September and October 2021. VEH: vaccine effectiveness against hospitalization.

As stated previously, VEH was estimated by comparing the prevalence of COVID-19 hospitalizations in the partially or fully vaccinated group to that in the unvaccinated group. Figure 1 shows all VEH estimates with 95% CIs.

The VEH for full vaccination ranges between 0.90 and 0.98 in the 3 age groups and 6 time points, with a statistically significant ascending time trend in the youngest group. In contrast, the VEH for partial vaccination significantly declined during the study period, especially in the older age group. The average weekly decline is 0.01, 0.02, and 0.05 in the 3 age groups, respectively. The partial vaccination effectiveness estimates ranged from 0.52-0.77 among older adults and from 0.71-0.84 among the other adult groups. Estimates of VEH for partial vaccination are substantially less precise than that for full vaccination, as shown by the wider confidence interval, especially in the older adult group.

Table 2 shows the summary indicators of VEH obtained after pooling the data from the 6 observed time periods. These estimates represent the status of the COVID-19 vaccination effort in early October 2021 in Costa Rica. The age-adjusted estimates for all adults suggest a VEH of 93.4% (95% CI 93.0-93.9) for the full vaccination schedule of 2 doses and 77% (95% CI 75.0-78.3) for partial vaccination with 1 dose. Older adults showed slightly lower VEH for full vaccination (92%, 95% CI 91.4-92.5) and substantially lower VEH for partial vaccination (64%, 95% CI 57.5-69.4) compared to the other age groups. The majority of the COVID-19 hospitalizations were probably caused by the Delta variant, which was dominant at the time of the study according to the Costa Rican genomic tracking system of the variants of concern (INCIENSA, unpublished data).

**Table 2:** Vaccine effectiveness against hospitalization for full and partial COVID-19 vaccination and relative risk of the unvaccinated being hospitalized by age group in Costa Rica in October 2021.

Age groups			Full vaccination		Partial vaccination
**Vaccine effectiveness against hospitalization (95% CI)**					
	20-39 years	0.949 (0.932-0.963)		0.801 (0.774-0.825)	
	39-57 years	0.947 (0.939-0.954)		0.792 (0.770-0.811	
	≥58 years	0.920 (0.914-0.925)		0.639 (0.575-0.694	
	All ≥20 years, crude	0.806 (0.795-0.816)		0.835 (0.824-0.846)	
	All ≥20 years, age adjusted^a^	0.934 (0.930-0.939)		0.767 (0.750-0.783)	
**Relative risk of hospitalization of the unvaccinated (95% CI)**					
	20-39 years	19.8 (14.7-26.7)		5.0 (4.4-5.7)	
	39-57 years	18.9 (16.4-21.7)		4.8 (4.3-5.3)	
	≥58 years	12.5 (11.6-13.4)		2.8 (2.4-3.3)	
	All ≥20 years, crude	5.1 (4.9-5.4)		6.1 (5.7-6.5)	
	All ≥20 years, age adjusted^a^	15.3 (14.2-16.4)		4.3 (4.0-4.6)	

^a^Mantel-Haenzel estimate.

Another metric for demonstrating vaccine effectiveness is by comparing the risk of being hospitalized between the unvaccinated and vaccinated, as reported in the second half of Table 2. These metrics may be more meaningful for laypeople. Table 2 shows that risk of hospitalization in the unvaccinated was 15.3 (95% CI 14.2-16.4) times higher than that in the fully vaccinated and 4.3 (95% CI 4.0-4.6) times higher than that in the partially vaccinated.

There were significant differences between the crude and age-adjusted estimates for the entire adult population (Table 2). Age was a significant confounder in these data. Older individuals had a much higher risk of being hospitalized and were more likely to be fully vaccinated than other adults. These two associations meant that for the all-age estimate, the crude VEH was substantially lower in the older adult group than in the other age groups and, thus, lower than its real magnitude, which was estimated by the simple method proposed by Mantel and Haenzel [11] in 1959 (ie, as a weighted average of age-specific figures). The crude all-age VEH for full vaccination was 81% compared to 93% when the age was adjusted.

### Sensitivity Analysis

**Figure 2:**
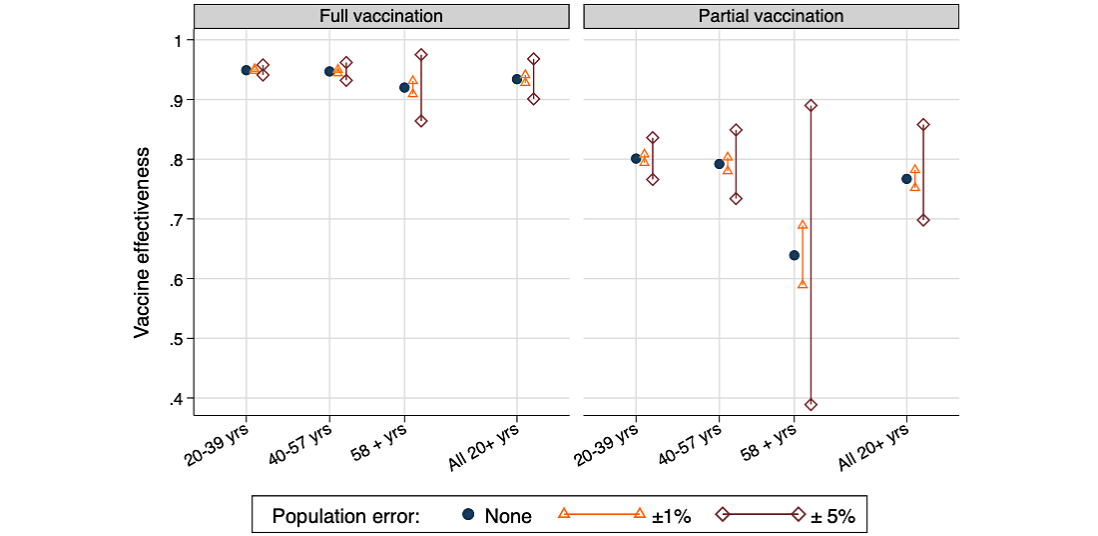
Sensitivity of estimates of vaccine effectiveness against hospitalization to possible errors in the population number used as input.

Figure 2 summarizes the sensitivity of VEH estimates to possible errors in the population data used as input. Errors of plus or minus 1% in the population input would bias the VEH estimates by less than 0.01 except in partially vaccinated older adults, where a change of 0.05 would occur. Larger errors of plus or minus 5% in the population input would alter the VEH by 0.02 in the two younger groups and would strongly bias the estimate between 0.04 and 0.27 for the partially vaccinated. VEH estimates for older adults appear especially sensitive to errors in the population data, which originates from the very high vaccination coverage reached by this age group: approximately 90% fully and 3% partially vaccinated at the end of the study period. Small errors in population inputs substantially amplify the residual estimates of unvaccinated individuals when vaccine coverage is high.

## Discussion

### Principal Findings

COVID-19 vaccine effectiveness to prevent severe illness, as identified by the prevalence of hospitalizations, is 93% for the complete vaccination scheme of 2 doses in the adult population of Costa Rica. Among the subgroup of adults aged ≥58 years, VEH was slightly lower (92%) than that in the other subgroups. The corresponding VEH estimates for 1 dose were 77% for all adults and 64% for the older subgroup. Costa Rica uses two COVID-19 vaccines, Pfizer-BioNTech (78% of the vaccines administered) and Oxford-AstraZeneca (22% of the vaccines administered) [2]. The estimates in this article largely reflect vaccine effectiveness against the Delta variant since it was the dominant variant in the country during the study period (INCIENSA, unpublished data).

The high VEH for the single dose showed a statistically significant decline in time trend by 0.01 or 0.02 per week in adults aged 20-57 years and 0.05 per week in the older age group. It is important to note that the decline in the VEH did not occur for full vaccination. If it was caused by the spread of the Delta variant or by depletion of the immunity provided by the vaccine, the decline should have also occurred among the fully vaccinated. A plausible explanation is that the partially vaccinated may have comprised two types of people: those who are in the group temporarily while waiting for the second shot and those who intentionally avoid the second shot and behave less carefully to avoid contagion. The VEH decline might reflect an increased share of the second subgroup as complete vaccination coverage approaches 100%.

### Comparison With Prior Studies

This study’s estimate of 93% VEH for 2 doses of the Pfizer-BioNTech and Oxford-AstraZeneca vaccines is within the 90%-98% range of previous real-world estimates obtained in Israel, Ontario, the United States, California, and Qatar [3-7]. Only the studies performed in California and Qatar reported that most of the sample population had the Delta variant, as reported in this study.

The 77% VEH for a single dose of the vaccines is also within the range of 70%-91% found by studies performed in Ontario, England, and Scotland [4,8,9]. However, these previous estimates predated the Delta variant. This Costa Rican estimate is thus the first study that has reported that the Delta variant did not substantially reduce the effectiveness of a single dose of either the Pfizer-BioNTech or Oxford-AstraZeneca vaccine to prevent hospitalization. Costa Rican adults aged ≥58 years received only the Pfizer-BioNTech vaccine, while the vaccine mix for the remaining adults was 77% Pfizer-BioNTech and 23% Oxford-AstraZeneca at the time of this study.

### Strengths

A strength of this observational study is that it shows the effectiveness of COVID-19 vaccines in the real-world conditions of a middle-income country, which is beyond the hypercontrolled conditions of clinical trials. Further strengths include that this study reports on the vaccines’ effectiveness after the Delta variant became dominant and on the dose-specific effectiveness of the vaccines.

The secondary analysis of existing aggregated data in this study is an inexpensive design that can be broadly used to produce quick estimates for timely monitoring of vaccine effectiveness.

Being a nationwide study based on the entire adult population, it is free of issues regarding sampling bias and random errors derived from small sample sizes.

The outcome variable used in this study, being hospitalized due to COVID-19–related conditions, is a definite count that is mostly free of classification errors. A threat to its validity as a measure of severe COVID-19 infections could occur if some people have poor access to hospital care. However, this is not the case in the universal health care system of Costa Rica.

The statistics of dose-specific vaccinated people are probably accurate since the sole provider of vaccines in the country digitally records real-time information of every single vaccine administered. The database for this information is also used for inventory control purposes and for providing digital vaccination certificates to the population. If there were widespread errors, they would certainly be noticed by these other uses of the data.

### Limitations

This observational study has the well-known limitations of nonrandomized, nonblinded trials including selection biases, such as the early vaccination of older people in Costa Rica (which biased the crude VEH estimates, as shown in this study), and other confounders such as the risk-taking behavior modification of some individuals after vaccination. The VEH estimates in this study should be interpreted as associations between vaccination status and hospitalization rather than the true causal effects of vaccination.

Being a study based on aggregated data, instead of microdata, it does not offer an opportunity to understand how differences at an individual level can contribute to VEH or can bias the VEH estimate. Potential errors derived from this limitation are sometimes called “ecological fallacy.”

A more specific limitation of the method used in this study is that it requires high-quality data of the population count to obtain a valid estimate of the number of unvaccinated individuals. Errors in the population count affect the calculation of the number of people who have not been vaccinated, especially as the vaccination coverage approaches 100%, as is the case for older adults in Costa Rica. However, it must be noted that Costa Rica is considered to have accurate demographic data [14].

The lack of specific results for each brand of vaccine used in Costa Rica, as well as the lack of estimates of the vaccines’ effectiveness at preventing COVID-19, may also be limitations of the interpretation of these study results.

### Conclusions

The Costa Rican vaccination program, based on the Pfizer-BioNTech and Oxford-AstraZeneca vaccines, was highly effective at preventing COVID-19–related hospitalizations even after the Delta variant became dominant. Completing the 2-dose scheme clearly provides more protection than that provided by a single dose, and this result must always be the goal of vaccination policies. However, the data also show that even a single dose appears to provide some protection against the Delta variant, which is good news for people whose second dose of the Pfizer-BioNTech vaccine was postponed several weeks to more quickly increase the number of people who received a first dose.

Timely monitoring of vaccine effectiveness appears feasible with procedures that are analogous to those used in this study. It is important to continue the monitoring of vaccine effectiveness to detect eventual failures in the vaccination program and motivate the public by showing that vaccinations are having an impact.

## Abbreviations

CCSS: Costa Rican Social Security Fund (Caja Costarricense de Seguro Social)
INCIENSA: Instituto Costarricense de Investigación y Enseñanza en Nutrición y Salud
INEC: National Institute of Statistics and Censuses (Instituto Nacional de Estadística y Censos)
RR: relative risk
VEH: vaccine effectiveness against hospitalization
